# Dr. John Hale Turner

**Published:** 1914-11

**Authors:** 


Dr. John Hale Turner, Buffalo, 1869, died at his home in Holley, N. Y., September 23, aged 70, following fracture of the hip. He was President of the Orleans Co. Medical Society in 1902 and coroner of the county for 15 years. He had practiced in Holley for 45 years.

